# Evolution of ANT family and regulation of lint percentage by the GhAIL6-GhTPL1 module

**DOI:** 10.3389/fpls.2025.1723288

**Published:** 2025-12-05

**Authors:** Jiwen Xu, Xiaoping Chen, Junjie Du, Zhijuan Liu, Tianzhen Zhang

**Affiliations:** 1 The Advanced Seed Institute, Zhejiang Provincial Key Laboratory of Crop Genetic Resources, Plant Precision Breeding Academy, College of Agriculture and Biotechnology, Key Laboratory of Plant Factory Generation-adding Breeding, Ministry of Agriculture and Rural Affairs, Zhejiang University, Hangzhou, China; 2 Hainan Institute of Zhejiang University, Sanya, China

**Keywords:** cotton, GhAIL6, GhTPL1, lint percentage, seed index

## Abstract

**Introduction:**

The AINTEGUMENTA/AINTEGUMENTA-like (ANT/AIL) family belongs to the APETALA 2/ETHYLENE RESPONSE FACTOR superfamily and plays a key role in regulating numerous developmental processes. Only limited research has been conducted on the ANT family in upland cotton (*Gossypium hirsutum* L.), despite upland cotton’s global importance as a major source of renewable textile fibers. Elucidating the mechanism of ANT/AILs in cotton fiber development is crucial for the breeding of high-yield cotton.

**Methods:**

Using phylogenetic trees explored the evolution of the ANT family. Using RNA sequencing data analyzed the expression of ANT/AILs in different cotton tissues. Using yeast two-hybrid (Y2H), dual-luciferase complementation assay (LUC) and bimolecular fluorescence complementation (BiFC) explored the interaction protein with GhAIL6. Using CRISPR-cas9 technology explored the regulation of GhAIL6-GhTPL1 module.

**Results:**

The ANT family originated in Bryophytes. A rapid expansion of ANT members occurred in the common ancestor of angiosperms, with the total number remaining stable in both Monocots and Eudicots. Cotton contained 20 ANT genes, as a duplication event in its ancestors led to expansion of AIL6/AIL7. Editing of GhAIL6 and GhTPL1 in upland cotton demonstrated AIL6-TPL1 module to regulate seed index (SI) and lint percentage (LP). Y2H, LUC and BiFC supported the interaction between GhAIL6 and GhTPL1.

**Discussion:**

Overall, this study offered a novel insight into the evolutionary dynamics of ANT family members and length. Edited cotton lines of GhAIL6 and GhTPL1, LP was significantly higher than in the control group, and SI significantly lower than in the control group. This study provided a new GhAIL6-GhTPL1 module for high-yield cotton breeding.

## 1 Introduction

Over the past twenty years, a number of AINTEGUMENTA/AINTEGUMENTA-like (ANT/AIL) transcription factors have been documented in *Arabidopsis thaliana*. Genes of this family belong to the APETALA 2 (AP2) subfamily and are characterized by two conserved AP2 domains. In *A. thaliana*, the core genes of the ANT lineage comprise WRINKLED1 (*WRI1*), WRINKLED3 (*WRI2*), and WRINKLED4 (*WRI4*). These genes are involved in regulating seed fatty acid metabolism, activating cutin biosynthesis, and modulating drought stress tolerance (To et al., 2012). Overall, there are eight ANT/AIL family genes in *A. thaliana*, namely *ANT*, *AIL1*, *AIL2/BBM/PLT4*, *AIL3/PLT1*, *AIL4/PLT2*, *AIL5/PLT5*, *AIL6/PLT3*, and *AIL7/PLT7* (Klucher et al., 1996; Nole-Wilson et al., 2005; Mizumoto et al., 2009; Bui et al., 2017; Han et al., 2022). ANT transcription factors play a crucial role in floral development, with overexpression of *ANT* resulting in larger floral organs (Elliott et al., 1996; Mizukami and Fischer, 2000). *AIL2* is likewise essential in both embryo and endosperm development, and its ectopic expression can induce somatic embryogenesis in seedlings (Horstman et al., 2014; Chen et al., 2022). *AIL3* and *AIL4* regulate patterning of the *A. thaliana* embryo and post-embryonic root meristem, influencing proper root development (Aida et al., 2004; Zhao et al., 2023). Overexpression of *AIL5* induces ectopic formation of shoot meristems, cotyledons, and the first leaf (Prasad et al., 2011), while all three of *AIL5*, *AIL6*, and *AIL7* are expressed in *A. thaliana* lateral root primordia and regulate lateral root development (Du and Scheres, 2017).

ANT/AILs have also been widely described in other species. In *Oryza sativa*, *OsSMOS1* acts downstream of auxin to regulate cell proliferation, thereby indirectly influencing cell size (Aya et al., 2014; Hirano et al., 2017). In *Zea mays*, the ANT-like protein Glossy15 regulates differentiation of epidermal cells in leaves, while ZmANT1 regulates vascular development, chloroplast development, photosynthesis, and plant growth (Moose and Sisco, 1994, Moose and Sisco, 1996; Xu et al., 2017). In *Medicago truncatula*, MtANT maintains leaf area size by influencing cell proliferation (Wang et al., 2022). In *Gnetum parvifolium*, *GpANTL1* is expressed in leaf primordia, root tips, and young ovules (Yamada et al., 2008). *CmoANT1.2* in *Cucurbita moschata* is critical to graft union formation, facilitating the grafting of pumpkin to cucumber (Miao et al., 2021). EgAP2-1, a homolog of BBM in *Elaeis guineensis*, is predominantly expressed in proliferating tissues such as leaf primordia (Morcillo et al., 2007). In *Populus trichocarpa*, overexpression of *PtAIL1* induces formation of more adventitious roots, while its silencing delays adventitious root formation (Rigal et al., 2012). Finally, in *Vitis vinifera*, *VviANT* is involved in regulating berry size (Chialva et al., 2016).

The floral development of higher plants is of great significance to agriculture. Over the past decades, researchers have gain achievements on floral formation mechanisms. Floral meristems (FMs) are formed in inflorescence meristems (IMs) and subsequently develop into flowers (Thomson and Wellmer, 2019). In Arabidopsis, the auxin efflux carrier PIN-FORMED1 (PIN1) regulate auxin accumulation influencing flower development (Huang et al., 2010). The auxin response factor ARF5 is activated by auxin accumulation in IM, and recruits SPLAYED (SYD) protein and BRAHMA (BRM) protein (Johnson et al., 2015). The ARF5-SYD module influences the expression of ANT and AIL6 genes, regulating floral development (Yamaguchi et al., 2013). Cotton is a major source of renewable textile fibers utilized throughout the world. Since the sequencing of the *Gossypium hirsutum* genome in 2015 (Li et al., 2015; Zhang et al., 2015), research on its functional genomics has advanced rapidly. However, studies on the function and regulatory networks of the ANT family in cotton remain scarce. Recent study has shown that *GhANT* can target the GoPGF protein and thereby both regulate the formation of secondary metabolites in glandular cells and reduce the number of mesophyll gland cells in leaves (Long et al., 2025). *GhANT* also regulates cotton seeds in ovules (Liu et al., 2025). A previous genome-wide association study (GWAS) of cotton bolls and seed index (SI) traits in 318 cultivated *G. hirsutum* accessions identified a significant association signal in the region spanning 79.10-79.45 Mb on chromosome A02. In the TM-1 genome, this region contains 16 genes, of which only four harbor nonsynonymous mutations; the researchers ultimately determined a nonsynonymous mutation in *GH_A02G1708* to be significantly associated with SI and lint percentage (LP) (Fang et al., 2017). Notably, *GH_A02G1708* encodes an AIL6 protein. In TM-1, the 175th and 185th amino acids are asparagine and glycine, respectively, whereas in Zhong Mian Suo 12 (ZMS12), both are mutated to aspartic acid. Compared to TM-1, ZMS12 exhibits a lower SI and a higher LP, leading to the designation of the allele in ZMS12 as *GhAIL6^HLB^* and that in TM-1 as *GhAIL6^LLB^*.

Research on the ANT/AIL family has yielded substantial findings; however, the variation in the number of ANT family among green plants, as well as the effects of ANT/AILs on LP and SI in cotton, has rarely been reported. In this study, we collected proteomic data from 1,509 green plant species and interrogated the evolution of the ANT family, demonstrating that the number of ANT family underwent an expansion in the ancestor of angiosperms. We also found that GhAIL6 interacted with GhTPL1 to regulate LP and SI development. These findings enhance the understanding of the expansion in the number of ANT family and lay a foundation for further investigating the ANT family’s regulation of seed development and improving crop yield.

## 2 Materials and methods

### 2.1 Collection of sequenced species

In this study, sequencing data from various species were collected for the exploration and construction of gene families. Initially, the ONEKP dataset was downloaded, and proteomic data were obtained. Subsequently, genome data of green plants were gathered from the NCBI (https://www.ncbi.nlm.nih.gov/), Phytozome (https://phytozome-next.jgi.doe.gov/), and CNGB databases (https://db.cngb.org/). The longest transcripts were retrieved based on mRNA data, and corresponding proteomic data were acquired. In the event of redundancy, proteomic data from sequenced species were prioritized and used to replace those in the ONEKP dataset. A total of 1,509 proteomes from a wide range of green plants were collected, comprising a total of 37,055,965 proteins. This collection included proteomic data from 949 species in the ONEKP dataset and 560 species with complete genome sequences.

### 2.2 Identification of the ANT family

Eight ANT/AILs of *A. thaliana* (AtANT, AtAIL1, AtAIL2/AtBBM/AtPLT4, AtAIL3/AtPLT1, AtAIL4/AtPLT2, AtAIL5/AtPLT5, AtAIL6/AtPLT3, AtAIL7/AtPLT7) were used as query sequences to search for similar sequences within the collected proteomic data. The resulting sequences were then subjected to domain analysis to confirm presence of the AP2 domain. Specifically, the steps were as follows: Firstly, the DIAMOND tool was used to identify similar sequences (Buchfink et al., 2021). The command used was:

diamond makedb –in 1509Proteomics.fa –db database

diamond blastp –db database –query AtANTs.fa –out Result.txt –max-target-seqs 1 –evalue 0.001 –threads 10.

Next, the InterProScan tool was employed to identify conserved domains (Quevillon et al., 2005). The command used was:

interproscan.sh -i proteins.fasta -o result.tsv -f tsv -cpu 8 -appl Pfam.

### 2.3 Construction of phylogenetic trees

Phylogenetic trees were constructed using the full-length sequences of ANT/AIL proteins. The specific steps were as follows: The MAFFT (Katoh and Standley, 2013), Trimal (Capella-Gutiérrez et al., 2009), and FastTree (Price et al., 2009) tools were installed on a Linux system. MAFFT was used for multiple sequence alignment with the following parameters: –maxiterate 1000 –auto. The resultant aligned data were trimmed using Trimal (Capella-Gutiérrez et al., 2009) with the parameter -gappyout. Finally, the phylogenetic tree was constructed using FastTree (Price et al., 2009) with the “JTT” model.

### 2.4 Yeast two-hybrid experiment

The yeast two-hybrid assay was performed using the GAL4 system (Clontech, USA). Y2H vectors were constructed as follows: Sequences encoding GhAIL6^HLB^ (from variety Zhong mian suo12) (Fang et al., 2017) and GhAIL6^LLB^ (from variety TM-1) (Fang et al., 2017) were cloned into the pGBKT7 vector using homologous recombination to produce the vectors pGBKT7-GhAIL6^HLB^ and pGBKT7-GhAIL6^LLB^. Those vectors were co-transformed with the pGADT7 vector into the Y2HGold yeast strain (WeidiBio, Cat. #YCL1002), which was then cultured on selective medium lacking leucine (Leu) and tryptophan (Trp) for 48–72 hours (Coolaber, Cat. #PM2221). Single colonies were selected on SD/-Trp-Leu solid medium and subjected to PCR for identification. Positive colonies were transferred onto quadruple dropout medium (SD/-Trp-Leu-His-Ade) and incubated for 72–96 hours (Coolaber, Cat. #PM2111). Normal growth of colonies indicated self-activation of *GhAIL6^HLB^* and *GhAIL6^LLB^*.

### 2.5 Subcellular localization

Plasmids pBinGFP4-GhAIL6^HLB^ and pBinGFP4-GhAIL6^LLB^ were introduced into *Agrobacterium tumefaciens* GV3101 (WeidiBio, Cat. #AC1001) via the heat shock method, after which the transformed bacteria were cultured at 28 °C for two days and single colonies were selected for identification. For positive cultures, a 500 μL aliquot was added to 20 mL of liquid LB medium containing 50 μg/mL kanamycin and 50 μg/mL rifampicin and incubated overnight at 28 °C with shaking. Afterwards, the culture was centrifuged at 5,000 rpm for ten minutes at room temperature, and the supernatant was discarded. The bacterial pellet was resuspended in infection solution (10 nM MES-KOH, 10 mM MgCl_2_, 200 μM AS) and adjusted to an OD_600_ of approximately 1.0. The suspension was then kept in the dark at 28 °C for three hours.

For infiltration, *Agrobacterium tumefaciens* carrying *GhAIL6^HLB^-GFP*/*GhAIL6^LLB^-GFP* was injected into the abaxial side of transgenic tobacco leaves expressing the H2B-RFP (nuclear localization signal) fusion protein. The plants were then incubated under weak light conditions for 48–72 hours. Afterward, leaf sections were cut from the infected areas and fluorescence signals were observed using a Zeiss LSM880 confocal microscope (Zeiss, Jena, Germany). The excitation wavelength for GFP was 488 nm, with emission at 507 nm; the excitation wavelength for RFP was 532 nm, with emission at 588 nm. Transmission light images of the cells were also collected to clearly observe cell morphology.

### 2.6 Protein structure prediction

The protein structures of GhAIL6^HLB^ and GhAIL6^LLB^ were predicted using AlphaFold2 (Jumper et al., 2021) and visualized using PyMOL (https://pymol.org/).

### 2.7 Plant materials and growth conditions

The cotton material used for the growth experiment was TM-1 (*Gossypium hirsutum*, Texas Marker-1), with cotton seeds directly sown into the experimental field. Cotton was grown under natural conditions in an open-field environment. The experiment was conducted in two separate regions: one at the Zhejiang University Experimental Base, Yuhang District, Hangzhou City, Zhejiang Province (latitude 30.3752d N, longitude 119.8765e E), sown in May 2024 and harvested in October 2024; the other at Bolangqiao, Yazhou District, Sanya City, Hainan Province (latitude 18.3625d N, longitude 109.1458e E), sown in October 2024 and harvested in March 2025.

### 2.8 Dual-luciferase complementation assay

The coding sequence (CDS) of each of the two interacting proteins was fused to the N-terminal and C-terminal regions of luciferase, respectively, to construct fusion protein expression plasmids (GhAIL6-Nluc and GhTPL1-Cluc). *Nicotiana benthamiana* plants were grown in a plant growth chamber under conditions of 16 h light/8 h dark photoperiod and 25 °5 for 4–5 weeks. Bacterial suspensions containing GhAIL6-Nluc and GhTPL1-Cluc were mixed at a 1:1 volume ratio and then infiltrated into tobacco leaves. The treated tobacco plants were cultured under low light for 24–48 h to ensure sufficient expression of the fusion proteins in the tobacco leaves. A 1 mM solution of D-luciferin potassium salt (MedChemExpress, Cat. #HY-12591B) was prepared and uniformly sprayed onto the abaxial surface of the tobacco leaves. After dark treatment for 3–5 min, the fluorescence intensity was observed using the chemiluminescence imaging system (Tanon-5200).

### 2.9 Bimolecular fluorescence complementation assay

The CDSs of the two target proteins to be tested (GhAIL6 and GhTPL1) were cloned into the pSPYNE and pSPYCE vectors, respectively. Bacterial suspensions containing these two different plasmids were mixed at a 1:1 volume ratio and then infiltrated into tobacco leaves. The treated tobacco plants were cultured under low light for 24–48 h to ensure sufficient expression of the fusion proteins in the tobacco. Tissue samples near the infiltrated areas of the tobacco leaves were collected, and fluorescence imaging was performed using a Zeiss LSM880 confocal microscope (Zeiss, Jena, Germany).

### 2.10 High-throughput confirmation of gene-edited materials

Mutant materials of GhAIL6 and GhTPL1 were created using the CRISPR-Cas9 technology (Liu et al., 2017). Two pairs of sgRNAs were selected to target the exon regions of each of the two genes. The CRISPR-Cas9 gene-editing experiments were conducted by Wimi Biotechnology (Jiangsu, China). Identification of gene-edited plants was performed using the high-throughput sequencing (Liu et al., 2019). Plants of the T3 generation were used for subsequent experiments.

### 2.11 Investigation of agronomic traits

Field experiments were conducted in the experimental fields of Sanya (Hainan Province) and Hangzhou (Zhejiang Province). Homozygous gene-edited materials and recipient materials were planted in the same area, with three plot replications. During the harvest period, cotton bolls were collected from the middle part of each cotton plant in every row. These samples were evaluated in terms of LP and SI.

### 2.12 DNA affinity purification sequencing

DNA Affinity Purification Sequencing (DAP-seq) is a method that successfully transfers *in vivo* binding experiments to *in vitro* conditions (Bartlett et al., 2017). Expression vectors were constructed by adding protective bases to both ends of *GhAIL6^LLB^* and ligating them into the vector. The constructed vectors were used in a cell-free expression system in wheat germ extract kit (Promega, Cat. #L326A), and the expressed protein was detected by Western blot. The obtained GhAIL6^LLB^ protein was then incubated with a DNA library (Yeasen, Cat. # No.13577), PCR with indexed adapters was performed on the bound sequences, and the products were sequenced using an Illumina HiSeq sequencer (Yeasen, Cat. #12412ES02). Clean reads were aligned to the *G. hirsutum* genome, peak calling was performed using MACS2 (v2.1.7), and the generated output files were visualized using deepTools2 (Ramírez et al., 2016). Enriched functions of GhAIL6^LLB^-bound genes were determined using the free online data analysis platform OmicShare (http://www.omicshare.com/tools). Analysis of DAP-seq peaks primarily employed bioinformatics approaches, including motif enrichment analysis, overlap with open chromatin regions, and consistency among biological replicates. Statistical analyses utilized the chi-square test and Z-test.

### 2.13 Statistical analysis

Statistical tests assessing species differences in ANT family protein length and number utilized the Mann-Whitney U test. *P < 0.05, **P < 0.01, ***P < 0.001, ns *P* > 0.05. The analysis was performed in R.

Statistical analysis of differences in cotton lint percentage and seed index used Student’s *t*-test, followed by a two-tailed test for *post-hoc* analysis (*n* = 5). When the *P*-value was greater than 0.05, the compared groups were considered to have no statistically significant difference. The experiment was performed in three biological replicates. The analysis was performed using Excel 2021.

## 3 Result

### 3.1 Origin of the ANT family in green plants

**Figure 1:**
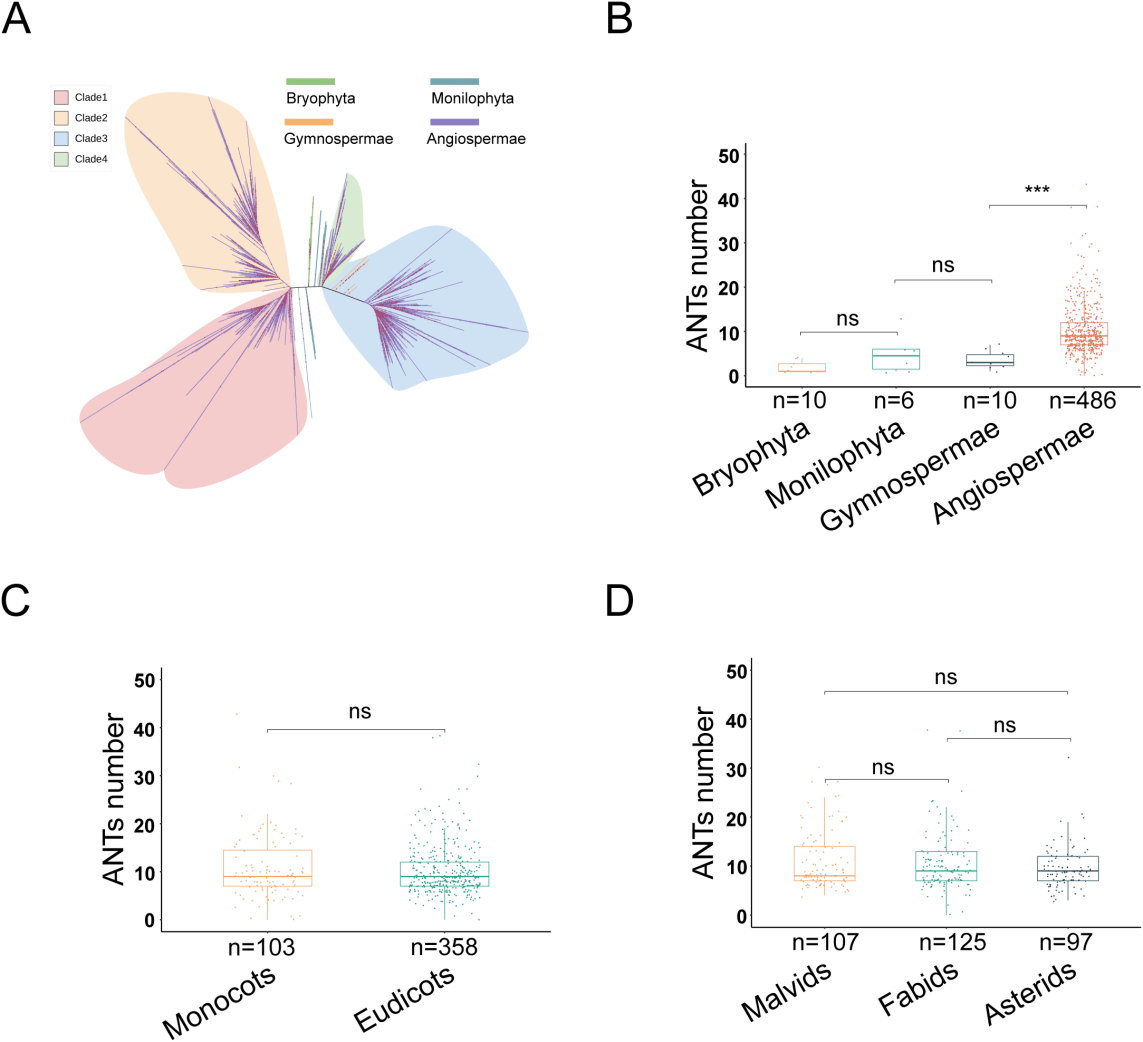
The evolution of ANT family in green plants. **(A)** Phylogenetic tree of 6,305 ANT proteins (ANTs). Colors represent different phyla. Bryophyta, Monilophyta, Gymnospermae, Angiospermae. **(B)** The number of ANTs in different phyla (Bryophyta, Monilophyta, Gymnospermae, Angiospermae) of terrestrial plants. **(C)** The number of ANTs in different class (Monocots and Eudicots) of Angiospermae. **(D)** The number of ANTs in Malvids, Fabids and Asterids of Eudicots.

A total of 6,305 ANT family genes were identified across the proteomes of 1,509 species, and their amino acid sequences were used to construct a rootless phylogenetic tree. The ANT family originated in green plants, with no homologous genes identified in metazoa and fungi. Within green plants, no ANT family members were identified in Chlorophyta or Charophyta. However, ANT/AILs were identified in Bryophyta, Monilophyta, Gymnosperms, and Angiosperms. In the phylogenetic tree, the branch of Bryophyta ANTs was positioned at the center, and evolution occurred in two directions, eventually forming Clades 1-4. Clades 1 and 2 were unique to Angiosperms, while Clades 3 and 4 included members in Gymnosperms (**Figure 1A**).

To explore variation in the number of ANT family genes within green plants, a statistical analysis of ANT number across 560 sequenced plant genomes was performed using the Mann-Whitney U test (**Supplementary Table 1**). There were no significant differences in gene number among the Bryophyta (*n* = 10, mean: 2, median: 1, standard deviation [SD]: 1), Monilophyta (*n* = 6, mean: 5, median: 5, SD: 4), and Gymnosperms (*n* = 10, mean: 4, median: 3, SD: 2) (**Figure 1B**; **Supplementary Table 2**). However, during the formation and evolution of Angiosperms (*n* = 486, mean:10, median: 9, SD: 6), a significant expansion of ANT genes evidently occurred, as Angiosperms exhibited a markedly higher number than Gymnosperms (Mann-Whitney U, *P* = 3.02e-6) (**Figure 1B**). These results indicated that the number of ANT genes underwent a significant expansion in the ancestors of angiosperms.

To further investigate the dynamic changes in the ANT family among Angiosperms, Monocots and Eudicots were similarly analyzed. The results showed no significant difference in ANT gene number (Monocots, *n* = 103, mean: 11, median: 9, SD: 7; Eudicots, *n* = 358, mean: 10, median: 9, SD: 5) (**Figure 1C**; **Supplementary Table 3**). An additional analysis was performed within Eudicots, which included groups such as Malvids, Fabids, and Asterids, each consisting of multiple orders. This revealed no significant difference in the number of ANT family genes between Malvids (*n* = 107, mean: 11, median: 8, SD: 6), Fabids (*n* = 125, mean: 11, median: 9, SD: 6), and Asterids (*n* = 97, mean: 10, median: 9, SD: 4) (**Figure 1D**; **Supplementary Table 4**). Taken together, these findings suggested that the expansion of the ANT family likely occurred in the common ancestor of Angiosperms, with gene number remaining relatively stable thereafter in both Monocots and Eudicots.

### 3.2 Evolution of the ANT family

**Figure 2:**
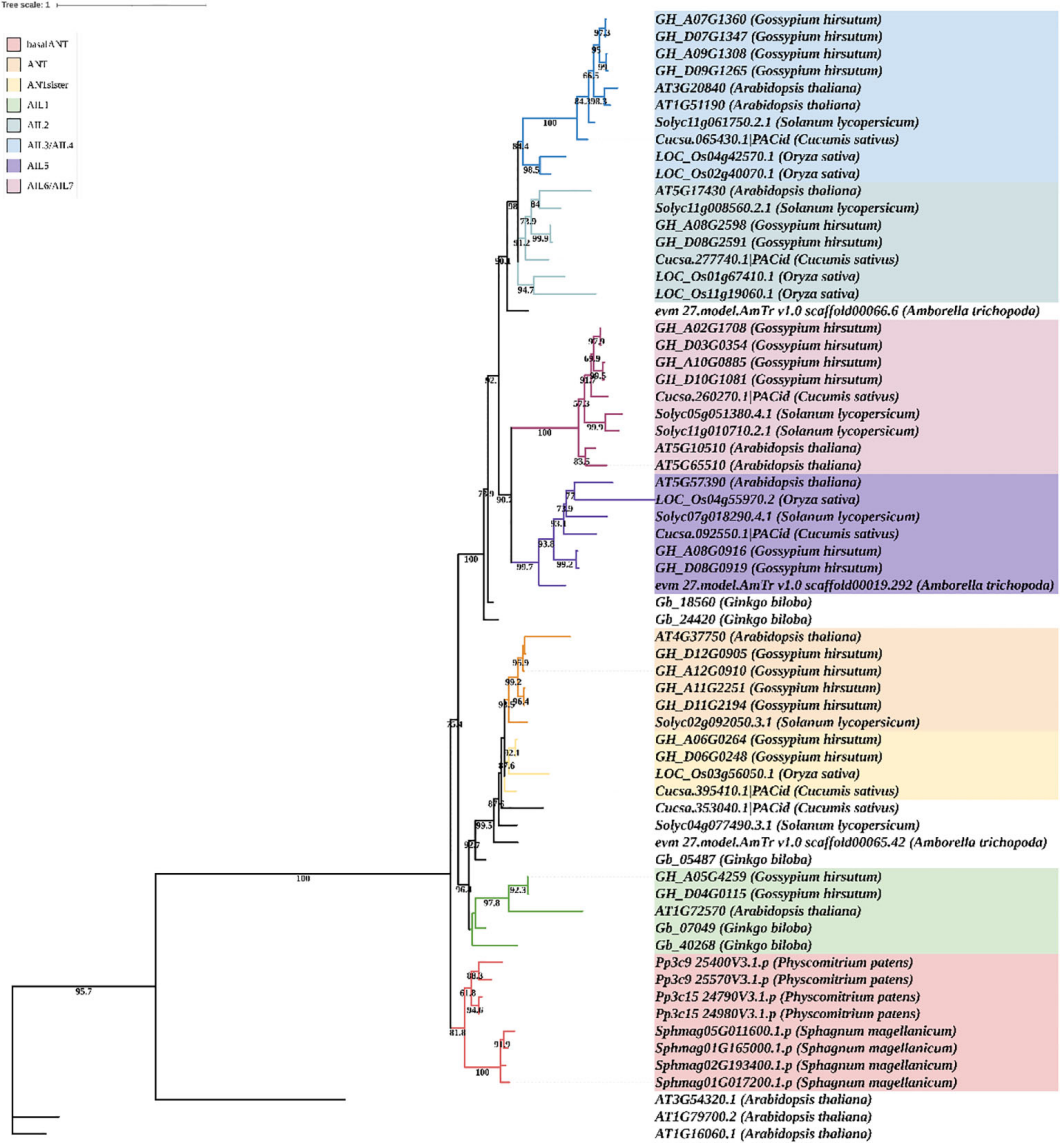
Phylogenetic tree of ANT/AILs in representative species (Physcomitrium patens, Sphagnum magellanicum, Ginkgo biloba, Amborella trichopoda, Oryza sativa, Arabidopsis thaliana, Gossypium hirsutum, Cucumis sativus, and Solanum lycopersicum. Label consists of gene id and species name. Colors represent different subfamilies. Bootstrap (> 70) is marked at tree.

To explore the evolution of the ANT family, a phylogenetic tree was constructed based on the ANT/AILs of *Physcomitrium patens*, *Sphagnum magellanicum*, *Ginkgo biloba*, *Amborella trichopoda*, *Oryza sativa* (rice), *A. thaliana*, *G. hirsutum* (cotton), *Cucumis sativus* (cucumber), and *Solanum lycopersicum* (tomato). Three WRI proteins (WRI1, WRI2, and WRI3) from *A. thaliana* were selected as the outgroup (**Figure 2**). The ANT/AILs in *P. patens* and *Sphagnum magellanicum* formed a distinct clade, representing the ancestor group of land plants, which was named “basalANT”. The ANT, ANTsister, and AIL1 subfamilies all originated from the common ancestor of Gymnosperms and Angiosperms, with the AIL1 subfamily diverging earlier than the other two. *G. hirsutum*, *A. thaliana*, and *Gingko biloba* included proteins of the AIL1 subfamily, while *O. sativa*, *Cucumis sativus*, and *Solanum lycopersicum* had lost AIL1. The divergence between the ANT and ANTsister subfamilies occurred in the common ancestor of Eudicots and Monocots. Cotton contained both ANT and ANTsister proteins; Arabidopsis and tomato possessed only ANT proteins, while rice and cucumber retained only ANTsister proteins (**Figure 2**). AIL2, AIL3/AIL4, AIL5, and AIL6/AIL7 originated from the common ancestor of basal Angiosperms, with which began the divergence of AIL5 and AIL6/AIL7. Arabidopsis, rice, cotton, cucumber, and tomato all possessed AIL5 genes. AIL6/AIL7 genes were lost in rice, but retained in Arabidopsis, cotton, cucumber, and tomato. Interestingly, independent duplications of AIL6/AIL7 occurred in the ancestors of Arabidopsis, cotton, and tomato, resulting in three distinct expansions of AIL6/AIL7 genes. The divergence of AIL2 and AIL3/AIL4 occurred in the common ancestor of Monocots and Eudicots, and both subfamilies were broadly retained across both groups. Two duplication events in the ancestor of rice led to the expansion of AIL2 and AIL3/AIL4 proteins in that species. A single duplication event in the ancestors of Arabidopsis and cotton resulted in the amplification of AIL6/AIL7 proteins in both species (**Figure 2**). These results indicated that members of the ANT/AIL family exhibited both expansion and loss in higher plants.

### 3.3 Evolution of ANT/AILs length

**Figure 3:**
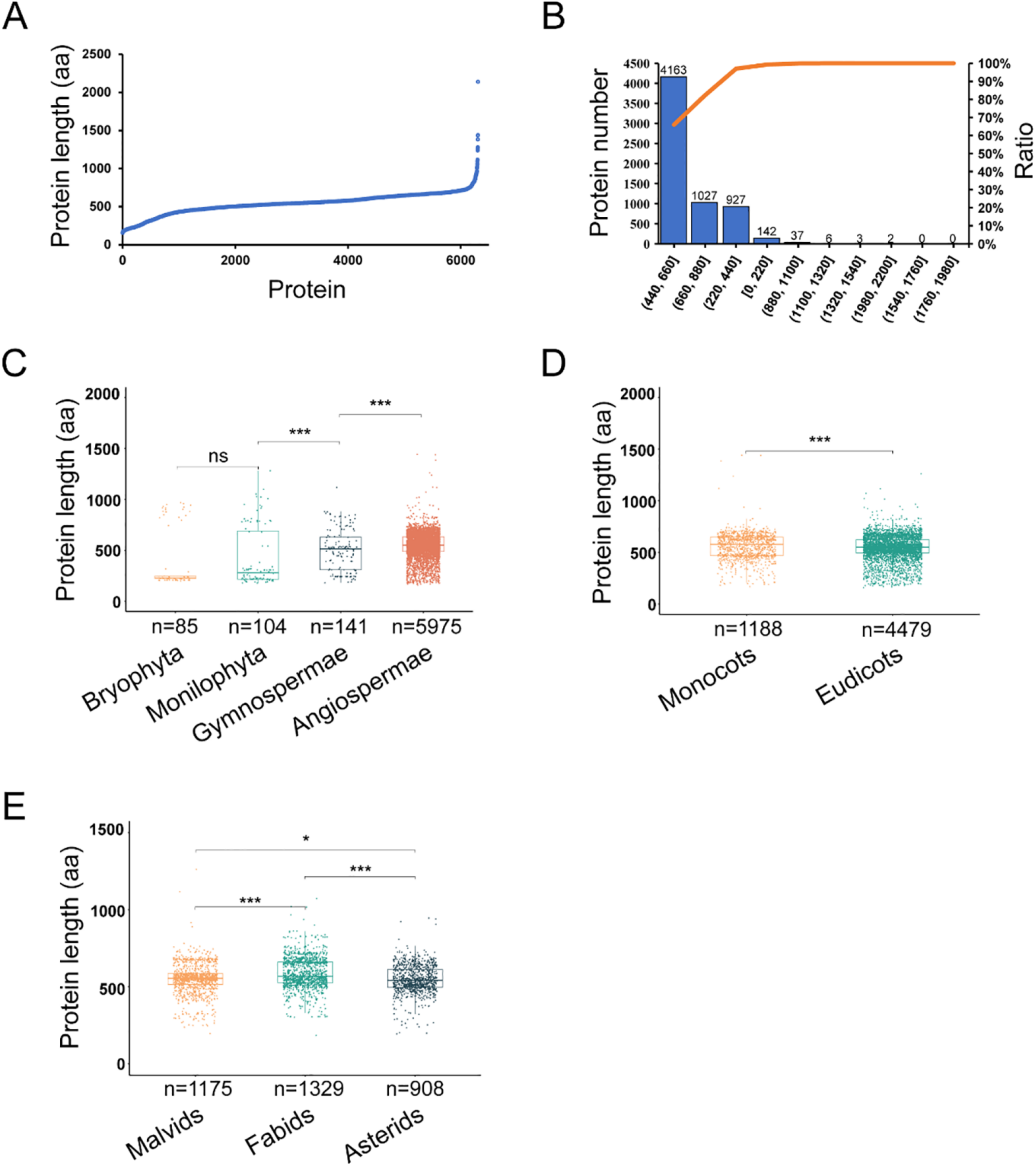
The evolution of 6,305 ANTs length in green plants. **(A)** The distribution of ANTs length. **(B)** The frequency distribution of ANT protein lengths. **(C)** The ANTs length in different phyla (Bryophyta, Monilophyta, Gymnospermae, Angiospermae). **(D)** The ANTs length in Angiospermae (Monocots and Eudicots). **(E)** The ANTs length in Eudicots (Malvids, Fabids and Asterids). *P< 0.05, **P < 0.01, ***P < 0.001, ns P > 0.05 by Mann-Whitney U test.

We next explored the variation in the length of ANT/AILs throughout evolution. Overall, the 6,305 ANT/AILs ranged from 159 to 2,141 amino acids in length (**Figure 3A**). The majority (66.0%) had lengths between 440 and 660 amino acids, while 16.3% fell within the 660 to 880 amino acid range, and 14.7% had lengths between 220 and 440 amino acids (**Figure 3B**). Clades showed significant differences in ANT/AIL length, namely the Monilophyta (*n* = 104, mean: 421, median: 281, SD: 285), Gymnosperms (*n* = 141, mean: 501, median: 515, SD: 200), and Angiosperms (*n* = 5975, mean: 548, median: 552, SD: 121) (**Supplementary Table 5**). The longest average and median lengths were observed in Angiosperms (**Figure 3C**). Results indicated that ANT/AILs proteins in angiosperms had the longest length.

Length variation in ANT/AILs within Angiosperms was further investigated through comparison of Monocots and Eudicots. A significant difference was found, with Eudicots (*n* = 4479, mean: 543, median: 549, SD: 121) having shorter ANT/AILs compared to Monocots (*n* = 1188, mean: 569, median: 588, SD: 116, Mann-Whitney U, *P* = 4.35e-4) (**Figure 3D**; **Supplementary Table 6**). Further analysis within Eudicots, specifically the Malvids, Fabids, and Asterids, revealed the longest ANT lengths to be present in Fabids (*n* = 1329, mean: 588, median: 568, SD: 97), followed by Malvids (*n* = 1175, mean: 553, median: 555, SD: 104), and the shortest in Asterids (*n* = 908, mean: 548, median: 541, SD: 89) (**Figure 3E**; **Supplementary Table 7**). Overall, it was clear that the length of ANT/AIL family members had dynamically evolved throughout plant evolution.

### 3.4 Expression of ANT/AILs in cotton

**Figure 4:**
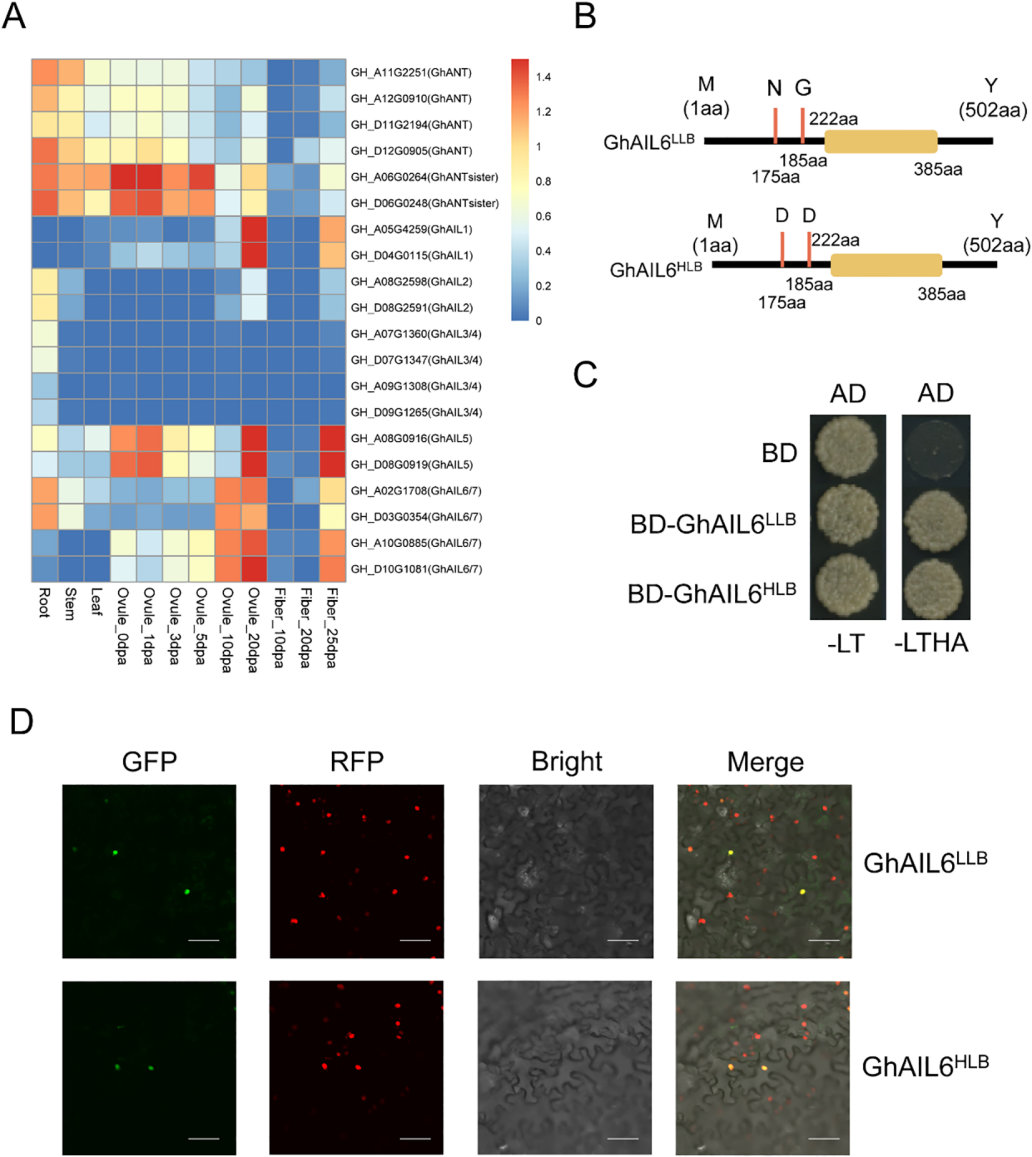
The ANT/AILs in upland cotton. **(A)** Expression pattern of ANT protein in different tissues of upland cotton. Root stands for root, Stem stands for stem, Leaf stands for leaf, Ovule stands for ovule, Fiber stands for fiber, and dpa stands for Days Post Anthesis, which represents the number of days after flowering. **(B)** Amino acid differences between the two proteins GhAIL6^LLB^ and GhAIL6^HLB^. **(C)** Verification of the self-activation ability of the two proteins GhAIL6^LLB^ and GhAIL6^HLB^. AD: pGADT7 vector, BD: pGBKT7 vector, -LT: SD/-Leu-Trp, -LTHA: SD/-Leu-Trp-His-Ade. **(D)** Subcellular localization of the two proteins GhAIL6^LLB^ and GhAIL6^HLB^. GFP: green fluorescent protein, RFP: red fluorescent protein, Bright: bright field, Merge: merged bright and dark field. The constructed GhAIL6^HLB^-GFP fusion protein and GhAIL6^LLB^-GFP fusion protein were transiently expressed in tobacco stably transformed with 35S:H2B-RFP. The scale bar in the figure represents 50 μm.

Leveraging previously published transcriptome data from *G. hirsutum* (http://cotton.zju.edu.cn/), the expression of 20 ANT/AILs was determined in various tissues, including roots, stems, leaves, and ovules and fibers at different days post anthesis (dpa) (**Figure 4A**). Two ANTsister genes showed relatively high expression in ovules at 0 dpa, 1 dpa, and 5 dpa. Two GhAIL1 genes had higher expression in ovules at 20 dpa and fibers at 25 dpa, with the highest expression in ovules at 20 dpa. Two GhAIL2 genes were expressed in roots, stems, ovules at 20 dpa, and fibers at 25 dpa. Four GhAIL3/GhAIL4 genes were expressed exclusively in the roots, with no expression detected in other tissues. GhAIL5 and GhAIL6/GhAIL7 genes were expressed in ovules at 10 dpa, 20 dpa, and in fibers at 25 dpa. Overall, GhANT/GhAILs exhibited some level of expression across the examined tissues.

Conserved domain analysis of the GhAIL6 protein revealed it to contain the AP2 domain, spanning amino acids 222 to 385; this domain is a conserved region in transcription factors known for its DNA-binding ability. The two nonsynonymous mutations distinguishing GhAIL6^HLB^ and GhAIL6^LLB^ are located in the non-conserved region upstream of the AP2 domain, even as the domain itself remains unchanged, suggesting that the DNA-binding capability of these proteins is not affected (**Figure 4B**). GhAIL6LLB and GhAIL6HLB proteins were cloned into BD plasmids and co-transformed into yeast cells along with AD plasmids. The transformed yeast grew normally on both double dropout medium (SD/-Leu-Trp) and quadruple dropout medium (SD/-Leu-Trp-His-Ade), indicating that both GhAIL6LLB and GhAIL6HLB exhibit self-activation activity—a key functional characteristic of transcription factors (**Figure 4C**). To investigate whether the mutations affect the localization of GhAIL6, GhAIL6^HLB^-GFP and GhAIL6^LLB^-GFP fusion proteins were transiently expressed in *Nicotiana benthamiana* stably transformed with 35S:H2B-RFP. The results showed both GhAIL6^HLB^ and GhAIL6^LLB^ to localize to the nucleus, hence their localization was not altered (**Figure 4D**). Together, these findings indicated that GhAIL6^HLB^ and GhAIL6^LLB^ retained transcriptional activation capability and were localized to the nucleus, where they likely participated in transcriptional regulation.

Rather than affecting DNA-binding ability, the mutations at amino acids 175 and 185 may influenced the ability of GhAIL6 to interact with its binding partners. Structural predictions of the GhAIL6^HLB^ and GhAIL6^LLB^ proteins using AlphaFold2 revealed the 175th and 185th amino acids to be located in a linear region with no distinct α-helix or β-sheet structures (**Supplementary Figure 1**) and to not exhibit any special local structural features (**Supplementary Figures 1A, B**). However, substitution of these positions to aspartic acid allowed the formation of hydrogen bonds with neighboring amino acids (**Supplementary Figures 1C, D**).

### 3.5 GhAIL6 regulates cotton lint percentage and seed index

**Figure 5:**
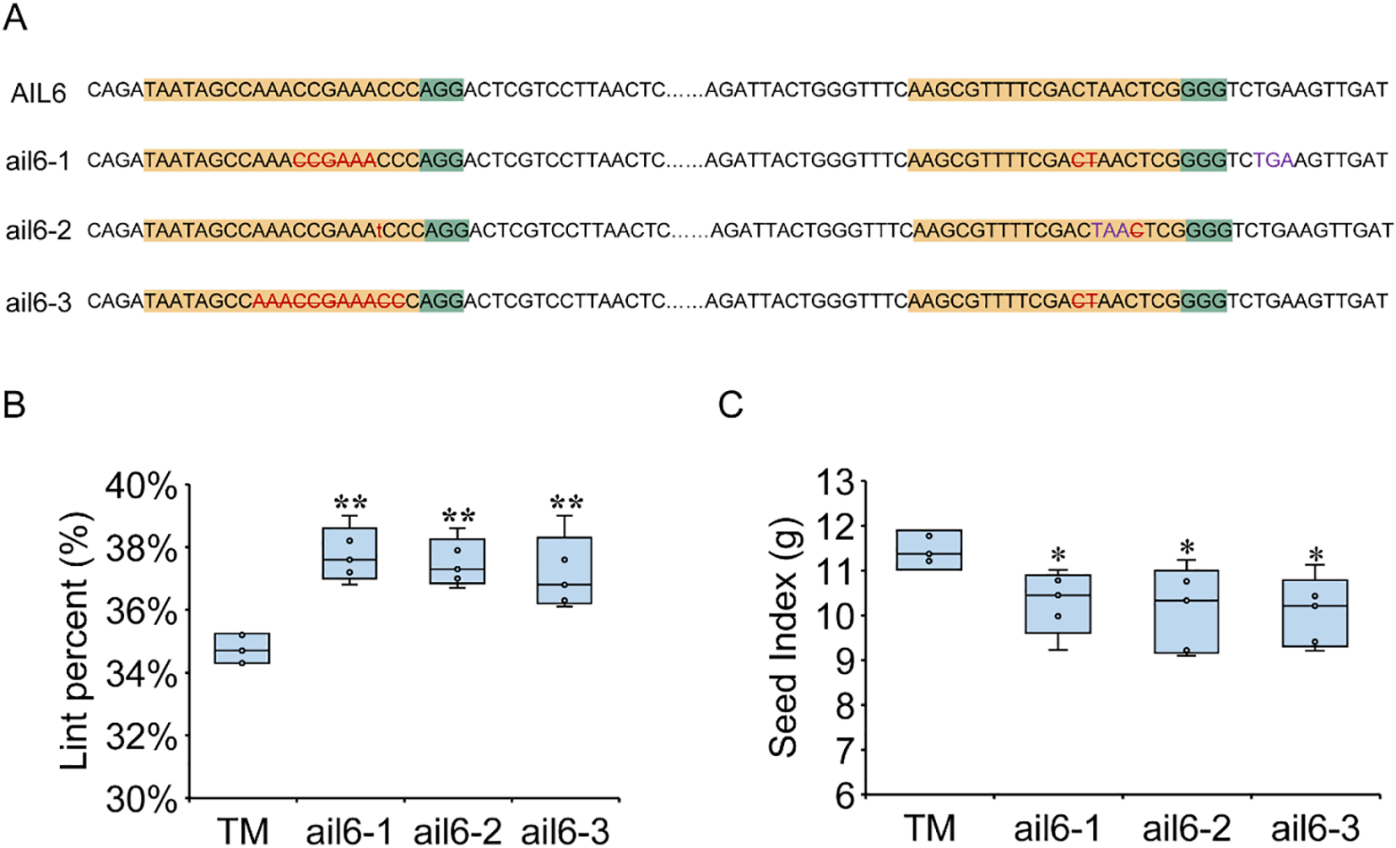
GhAIL6 regulates lint percentage and seed index of upland cotton. **(A)** Editing target and editing type of GhAIL6. Yellow represents the target sequence, green represents PAM, red represents the type of gene editing, purple represents the stop codon formed after gene editing, the number represents the position of the base on the CDS sequence, AIL6 represents the amino acid sequence in the TM-1 material, and ail6-1, ail6–2 and ail6–3 represent the three gene editing materials, respectively. **(B)** Comparison of TM-1, ail6-1, ail6–2 and ail6–3 lint percentage. **(C)** Comparison of TM-1, ail6-1, ail6-2, and ail6–3 seed index. *P < 0.05 and **P < 0.01 by two-tailed Student’s t-test (*n* = 5).

To investigate the effects of GhAIL6 on LP and SI, gene-edited materials were developed using TM-1 as the recipient. Two target sites were selected within the 302 bp to 468 bp region of *GhAIL6*, and three edited lines were obtained. Line ail6–1 had a six-base deletion at the first target site and a two-base deletion at the second, leading to the premature appearance of a stop codon (“TGA”). Line ail6–2 had a one-base insertion of “T” at the first target site, resulting in the premature occurrence of a stop codon (“TAA”). Line ail6–3 had an 11-base deletion at the first target site and a two-base deletion at the second, which disrupted the original amino acid coding sequence (**Figure 5A**). Cotton harvested from mature plants yielded a LP of 34.76 ± 0.42% in the control group (TM-1), 37.74 ± 0.77% in line ail6-1, 37.51 ± 0.67% in line ail6-2, and 37.18 ± 1.07% in line ail6-3. For all three edited lines, LP value was significantly higher than in the control group (**Figure 5B**). Conversely, SI value was 11.44 ± 0.42 g in the control group (TM-1), 10.29 ± 0.63 g in line ail6-1, 10.13 ± 0.84 g in line ail6-2, and 10.08 ± 0.70 g in line ail6-3 (**Figure 5C**), with all three *GhAIL6*-edited lines significantly lower than the control group (*P* < 0.05). These results indicated that GhAIL6 regulates the development of seed index and lint percentage.

### 3.6 Interaction between GhAIL6 and GhTPL1

**Figure 6:**
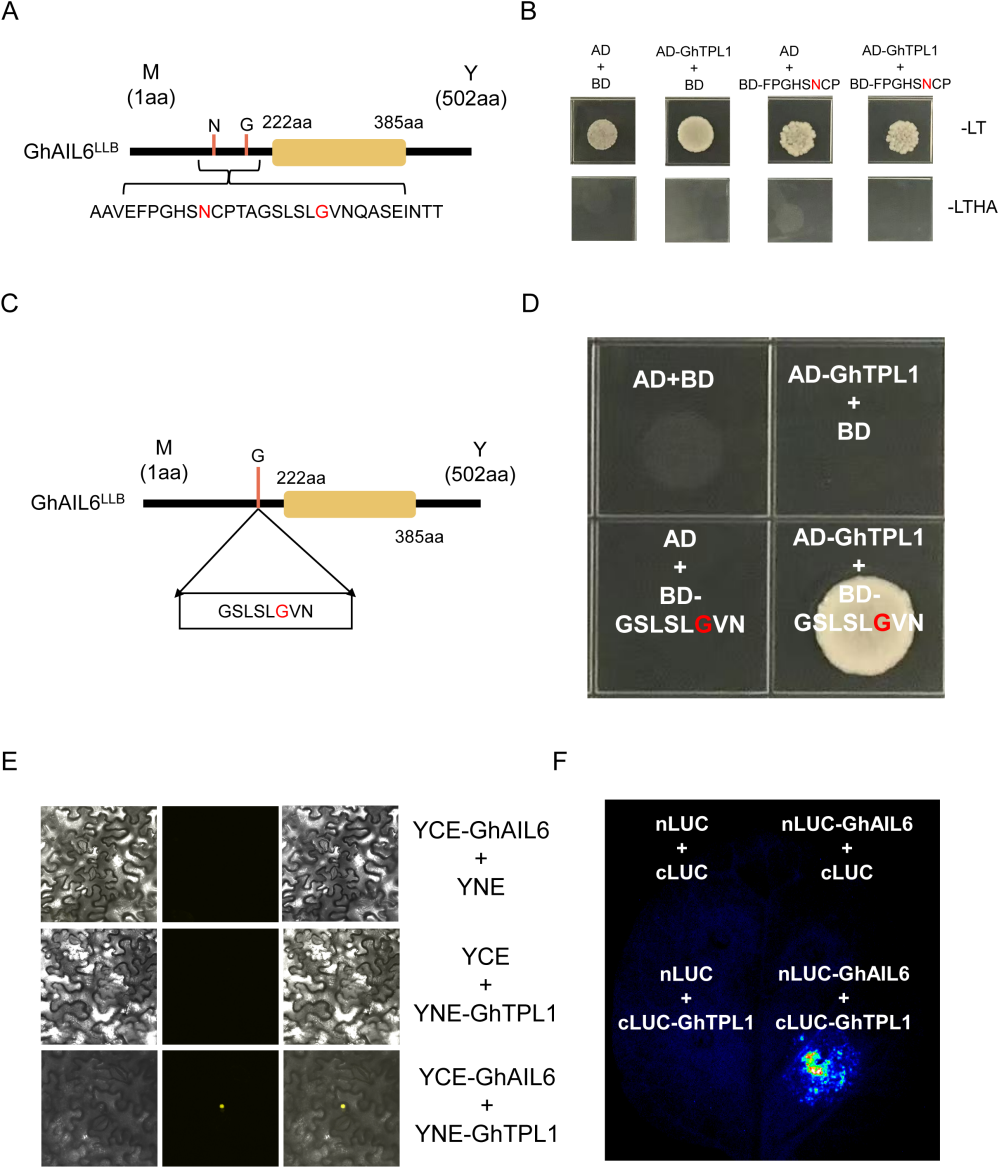
GhAIL6 interacts with GhTPL1. **(A)** 30 amino acid motifs containing two sites of variation. **(B)** Yeast two-hybrid experiment with GhTPL1 protein of the first motif. AD: pGADT 7 vector, BD: pGBKT 7 vector, -LT: SD/-Leu-Trp, -LTHA: SD/-Leu-Trp-His-Ade. **(C)** The second motif location and variant type of GhAIL6 protein. **(D)** Yeast two-hybrid experiments with GhTPL1 of the second motif performed on SD/-Leu-Trp-His-Ade medium. AD: pGADT 7 vector, BD: pGBKT 7 vector. **(E)** Double-luciferase complementation assay of GhAIL6 and GhTPL1. **(F)** Bimolecular fluorescence complementation experiment of GhAIL6 and GhTPL1.

To investigate the interacting proteins of the GhAIL6 protein, the Y2H assay was used, which identified the GhTPL1 protein as a potential interacting partner of GhAIL6. Firstly, validation was performed on the “FPGHSNCP” motif, where the 175th amino acid of GhAIL6 was located (**Figure 6A**). This “FPGHSNCP” motif was cloned into the BD vector, while the GhTPL1 gene was inserted into the AD vector. The two plasmids were then co-transformed into yeast cells. The co-transformed yeast cells grew normally on the double-dropout (SD/-Leu-Trp) medium but failed to grow properly on the quadruple-dropout (SD/-Leu-Trp-His-Ade) medium (**Figure 6B**). This result indicated that the first motif, “FPGHSNCP”, was unable to interact with the GhTPL1 protein.

Subsequently, the second motif, “GSLSLGVN” (**Figure 6C**), was cloned into the BD vector and co-transformed into yeast cells together with the AD vector harboring GhTPL1. Notably, this plasmid combination enabled the yeast cells to grow normally on the quadruple-dropout (SD/-Leu-Trp-His-Ade) medium, whereas the negative control did not support normal yeast growth (**Figure 6D**). Furthermore, both dual-luciferase complementation (LUC) assay and bimolecular fluorescence complementation (BiFC) assay were conducted, which further confirmed the interaction between the GhAIL6 protein and the GhTPL1 protein (**Figures 6E, F**). Collectively, these results demonstrated that GhAIL6 protein interacted with GhTPL1 specifically through its “GSLSLGVN” motif.

### 3.7 GhTPL1 regulates cotton lint percentage and seed index

**Figure 7:**
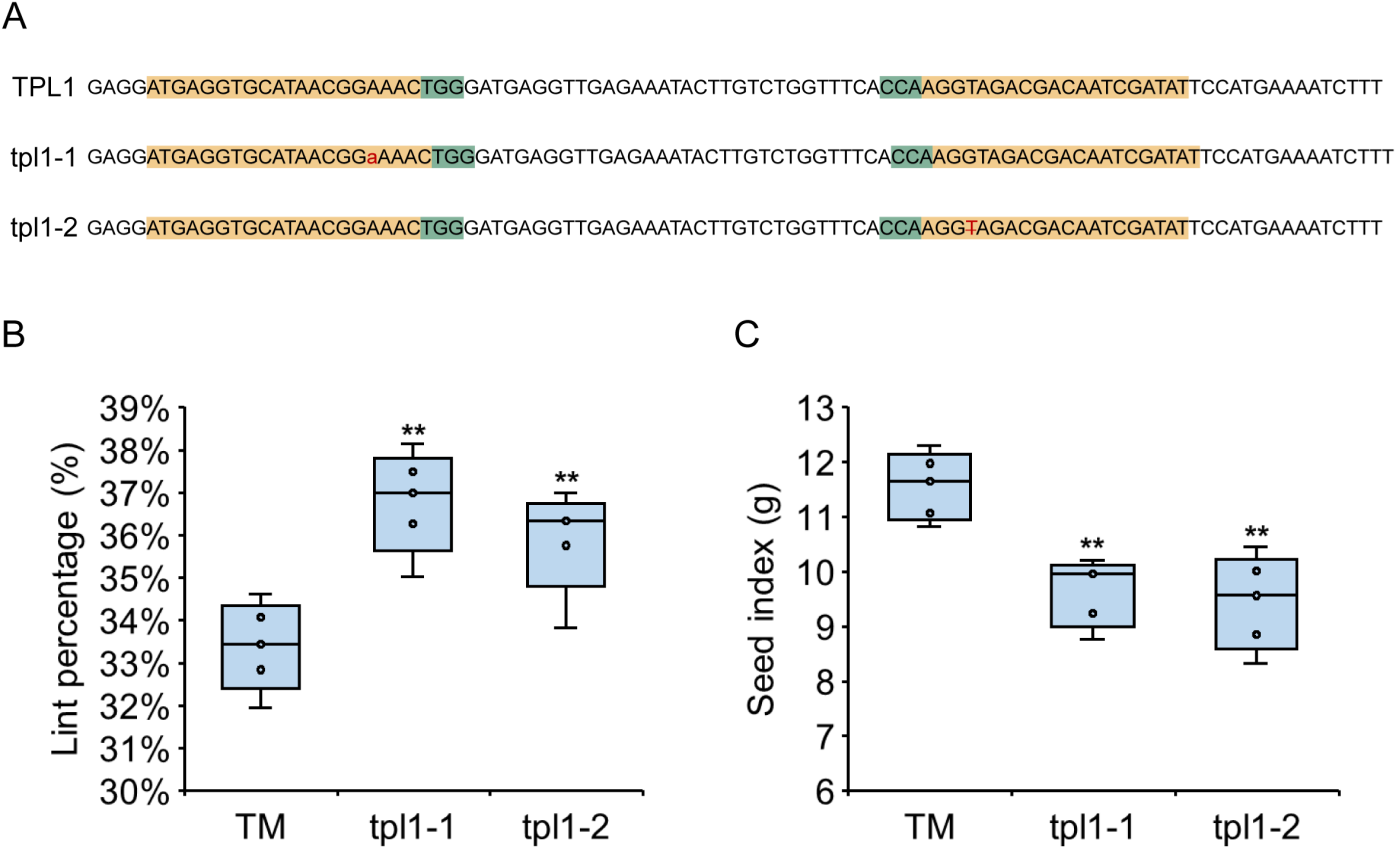
GhTPL1 regulates lint percentage and seed index of upland cotton. **(A)** Editing target and editing type of GhTPL1. Yellow represents the target sequence, green represents PAM, red represents the type of gene editing, purple represents the stop codon formed after gene editing, the number represents the position of the base on the CDS sequence, TPL1 represents the amino acid sequence in the TM-1 material, and tpl1–1 and tpl1–2 represent the three gene editing materials, respectively. **(B)** Comparison of TM-1, tpl1–1 and tpl1–2 lint percentage. **(C)** Comparison of TM-1, tpl1–1 and tpl1–2 seed index. *P < 0.05 and **P < 0.01 by two-tailed Student’s t-test (*n* = 5).

To investigate the effects of GhTPL1 on LP and SI, gene-edited materials were developed using TM-1 as the recipient. Two target sites were selected within the 124 bp to 219 bp region of *GhTPL1*, and two edited lines were finally obtained (**Figure 7A**). Line tpl1–1 had a one-base insertion of “A” at the first target site. Line tpl1–2 had a one-base deletion of “T” at the second target site. Cotton harvested from mature plants yielded a LP of 33.39 ± 0.94% in the control group (TM-1), 36.79 ± 1.07% in line tpl1-1, and 35.89 ± 1.09% in line tpl1-2. For all two edited lines, LP value was significantly higher than in the control group (**Figure 7B**). Conversely, SI value was 11.57 ± 0.55 g in the control group (TM-1), 9.64 ± 0.55 g in line tpl1-1, and 9.44 ± 0.77 g in line tpl1-2 (**Figure 7C**), with all two *GhTPL1*-edited lines significantly lower than the control group (*P* < 0.05). These results indicated that GhTPL1 was involved in regulating the development of seed index and lint percentage in cotton.

### 3.8 Identification of GhAIL6 downstream regulatory genes

GhAIL6, as a transcription factor, has an AP2 domain that can interact with cis-regulatory elements in the promoter regions of target genes. Since this domain was unchanged between GhAIL6^HLB^ and GhAIL6^LLB^, only the GhAIL6^HLB^ protein was used for the identification of candidate target genes. DAP-seq is a DNA affinity purification sequencing method that, compared to ChIP-seq, shifts *in vivo* detection to an *in vitro* setting and thereby increases the discovery rate of cis-regulatory elements bound by transcription factors (O’Malley et al., 2016). In brief, the GhAIL6 protein was fused with Halotag, and then incubated with a *G. hirsutum* genomic DNA library to capture DNA fragments. The bound fragments were then isolated, sequenced, and analyzed to identify the binding sites. The quality of the genomic DNA was presented in **Supplementary Figure 2A**. Genomic DNA was fragmented using a mechanical ultrasonic method, and the quality of the fragmented DNA was assessed by electrophoresis (**Supplementary Figure 2B**). For *in vitro* expression, the GhAIL6-Halotag fused sequence was cloned into a recombinant plasmid using the PCR-based Accurate Synthesis (PAS) method and expressed in a wheat germ cell-free protein expression system. The quality of expressed proteins was evaluated (**Supplementary Figure 2C**) and the fusion protein detected using Western blotting (**Supplementary Figure 2D**). Finally, the GhAIL6 fusion protein was incubated with the fragmented genomic DNA library, with the binding quality assessed by SDS-PAGE (**Supplementary Figure 2E**).

After quality control of sequencing data, 27,385,810 reads were obtained for the control group, alongside higher numbers of 36,551,260 and 43,187,436 respectively for experimental groups 1 and 2. The GC content of the control group was 36.49%, higher than in both experimental group 1 (36.08%) and experimental group 2 (36.04%) (**Supplementary Table 8**). Alignment of high-quality sequencing reads with the reference genome yielded alignment rates of 98.78% for the control group, 98.73% for experimental group 1, and 98.70% for experimental group 2. Thus, all three achieved a high alignment rate (**Supplementary Table 9**). The libraries were subsequently analyzed to determine the non-redundant fraction (NRF), PCR bottlenecking coefficient 1 (PBC1), and PCR bottlenecking coefficient 2 (PBC2). Both NRF and PBC1 were above 0.80 in all groups. The PBC2 for the control group was 8.81, while the corresponding values for experimental groups 1 and 2 were 5.61 and 4.98, respectively (**Supplementary Table 10**).

**Figure 8:**
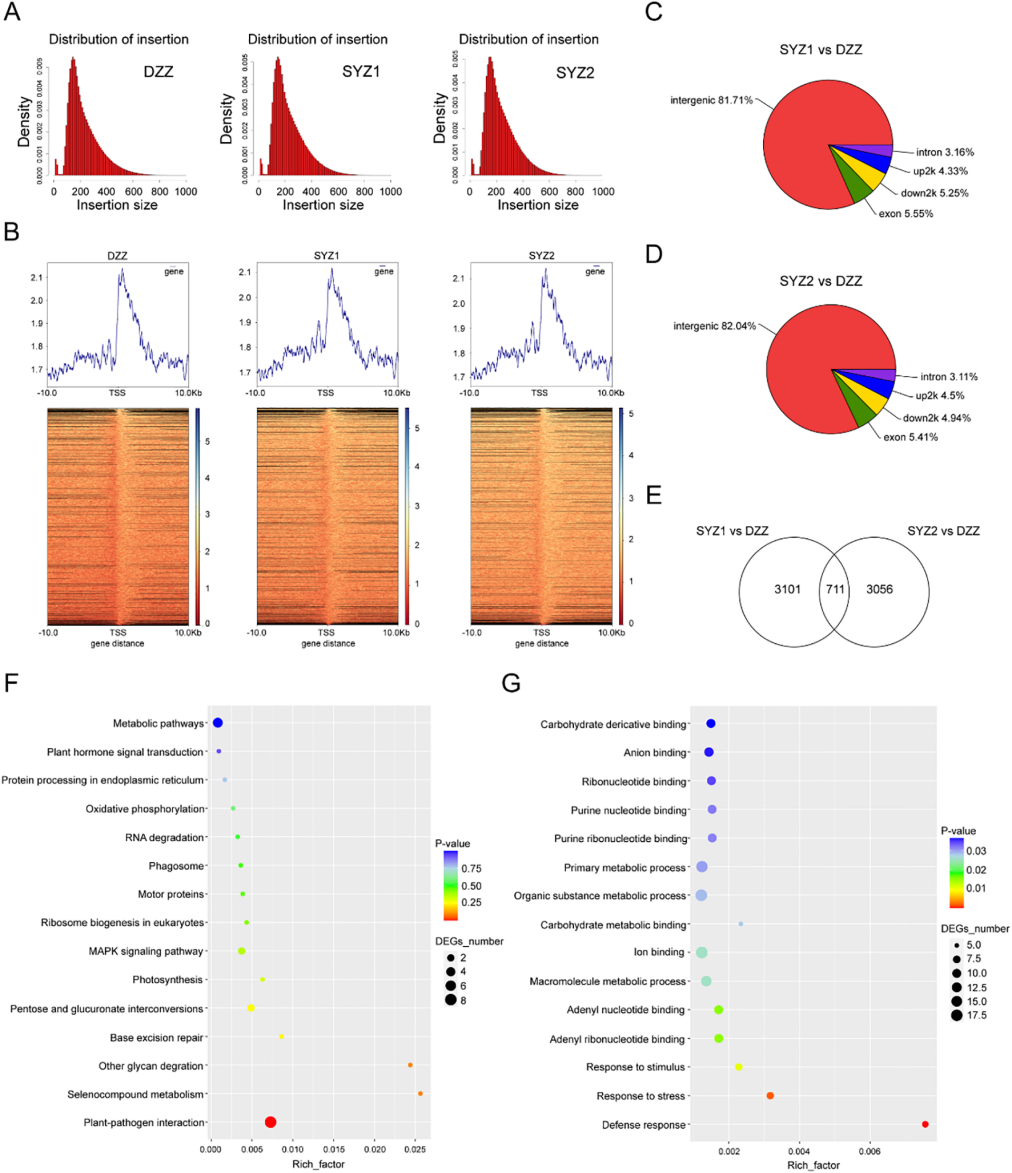
DAP-seq technology for finding the downstream genes and networks of GhAIL6. **(A)** Distribution of insert length in the control group (DZZ), experimental group 1 (SYZ1), and experimental group 2 (SYZ2). **(B)** Upstream and downstream abundance of different treatments in the body region of the gene. **(C)** Distribution of functional elements of Peak in SYZ1 vs DZZ. **(D)** Distribution of functional elements of Peak in SYZ2 vs DZZ. **(E)** Statistics of the intersection of the peaks of the two experimental groups. **(F)** GO enrichment analysis of 711 Peaks. **(G)** KEGG analysis of 711 Peaks.

**Figure 8A** presented the alignment of insert fragments for the control and experimental groups. In both groups, regions with higher average read abundance were primarily located between gene transcription start and termination sites (**Figure 8B**). These peaks indicated potential binding regions for the GhAIL6 protein. When comparing experimental group 1 with the control group, a total of 3,812 differential peaks were identified, with a total length of 1,377,221 bp. Similar comparison of experimental group 2 with the control group yielded 3,767 differential peaks, with a total length of 1,351,797 bp (**Supplementary Table 11**). Plotting the peak length distribution showed GhAIL6 binding peaks to predominantly span 200 to 400 bp (**Supplementary Figure 3**). With regard to the distribution among genomic regions, 81.71% of peaks were located in intergenic regions (experimental group 1) (**Figure 8C**). In experimental group 2, 82.04% of peaks were sited in intergenic regions (**Figure 8D**).

Intersection of the peak sets from the two experimental groups resulted in a total of 711 common peaks (**Figure 8E**). GO enrichment analysis of shared peaks revealed the genes which involved in processes such as carbohydrate derivative binding, anion binding, organic substance metabolism, carbohydrate metabolism, ion binding, macromolecule metabolism, response to stimuli, response to stress, and defense response (**Figure 8F**). KEGG pathway analysis indicated involvement in metabolic pathways, plant hormone signal transduction, protein processing in the endoplasmic reticulum, oxidative phosphorylation, MAPK signaling pathway, photosynthesis, and plant-pathogen interactions (**Figure 8G**). As with the respective total peak sets, the 711 peaks were widely distributed across intergenic, promoter, intronic, and exonic regions. The GhAIL6 protein was found to bind to 345 transient response elements within promoter regions, where these elements are distributed in the promoter regions of 24 genes (**Supplementary Table 12**).

## 4 Discussion

### 4.1 Expansion and loss of ANT family members in angiosperms

It was initially proposed that the ANT family originated in the common ancestor of bryophytes and ferns (Kim et al., 2006). However, with recent increases in sequenced species, ancient preANT genes have been identified in Charophyta; notably, these genes do not include the euANT and ANT families that are characteristic of embryophytes (Dipp-Álvarez and Cruz-Ramírez, 2019). This study revealed that ANT genes underwent duplication in the common ancestor of bryophytes and ferns, then subsequently evolved into two clades. These clades further diverged into four during the evolution of angiosperms—a finding consistent with previous reports. In previous studies of ANT family evolution, researchers typically selected a small number of representative species for analysis (Kim et al., 2006; Dipp-Álvarez and Cruz-Ramírez, 2019). In this study, we found that ANT gene number was maintained stably across monocotyledonous and dicotyledonous plants, and no obvious expansion or contraction was observed in dicots, specifically asterids, malvids, and fabids.

*AtAIL3* and *AtAIL4* act as downstream regulators of the jasmonic acid signaling pathway to modulate root development (Saiga et al., 2008). This study found a duplication of the AIL3/AIL4 subtribe also occurred in the ancestor of Arabidopsis. *AtAIL6* and *AtAIL7* show partial redundancy in regulating inflorescence and vegetative bud meristems (Mudunkothge and Krizek, 2012). Independent duplication events of the AIL6/7 subtribe were identified in the respective ancestors of tomato, Arabidopsis and cotton. The relative recent occurrence of duplication events results in the genes in question having high homology and partial conservation of their functions, thereby leading to partial functional redundancy.

### 4.2 The interaction between GhAIL6 and GhTPL1 represents a typical case of the interaction between EAR motifs and TPL proteins

Transcriptional repression mediated by the Ethylene Responsive Element Binding Factor Associated Amphiphilic Repression (EAR) motif is one of the major mechanisms regulating gene expression (Ohta et al., 2001). This motif (LxLxL or DLNxxP) has 6–8 amino acids, but is widely involved in regulating growth, development, and physiological metabolism (Yang et al., 2018). TPL family proteins consist of an N-terminal TPD domain and two C-terminal WD40 repeat sequences. The TPD contains three conserved domains: LisH, CTLH, and CT11-RanBPM (CRA). The TPD is conserved not only in sequence but also in structure (Plant et al., 2021). Substantial progress has been made in research on the interaction between EAR motifs and TPL/TPR. In *A. thaliana*, bHLH11 contains an LxLxL-type EAR motif, which helps plants maintain iron homeostasis by regulating the expression of iron deficiency genes (Li et al., 2022). In potato (*Solanum tuberosum*), a series of R2R3-MYB transcription factors containing LxLxL-type and DLNxP-type motifs is involved in metabolic and biochemical regulation (Li et al., 2020). In this study, the “FPGHSNCP” motif on GhAIL6 cannot interact with GhTPL1, whereas the “GSLSLGVN” motif (an EAR-like motif) can interact with GhTPL1.

Current research has focused on the interaction between EAR motifs and TPL/TPR proteins, which together regulate plant development. In several hormone signaling pathways, the EAR motif-TPL/TPR module also plays an indispensable role. TPL proteins play a role in repressing auxin-responsive genes and auxin primary response genes that bind to activated ARFs (Szemenyei et al., 2008). Similarly, this module is indispensable for regulating jasmonic acid (JA), strigolactones (SLs), Gibberellins (GAs), Brassinosteroids (BRs) signaling (Pauwels et al., 2010; Jiang et al., 2013; Wang et al., 2020; Fukazawa et al., 2015; Ryu et al., 2014). The AIL6 protein is a downstream regulator of ethylene signaling, and the GhAIL6-GhTPL1 module identified in this study represents a novel module in the ethylene signaling pathway.

### 4.3 The GhAIL6-GhTPL1 module plays a crucial role in regulating seed size

Seed sizes affect crops evaluation and crop yield. *AtAP2* regulates seed sizes and enlarges outer integument cells (Jofuku et al., 2005). The sweet cherry AP2/ERF transcription factor *PavRAV2* negatively regulates fruit size by directly repressing the expression of *PavKLUH* (Qi et al., 2023). *SlDREB3*, a member of the AP2/ERF transcription factor family, regulates seed size by integrating the ABA signaling pathway (Gupta et al., 2022). In rice, the OsEIL1-OsERF115 target gene regulatory module controls grain size and weight (Liu et al., 2022). TPL was first identified in the context of plant embryonic development along the polar axis. A recent study in rice demonstrates that OsNAL1 interacts with OsTPR2 to increase rice yield (Li et al., 2023). In this study, knockout of GhAIL6 and GhTPL1 reduced SI by 10% and 16.7%, respectively, which supported the involvement of the GhAIL6-GhTPL1 module in the regulation of SI.

### 4.4 The GhAIL6-GhTPL1 module regulates MAPK signaling pathway to influence seed index and lint percentage

Previous studies have mostly focused on the genetic interactions of ANT/AILs and their functions in development (Horstman et al., 2014). ANT/AIL proteins possess a typical AP2 domain that can bind to cis-acting elements and directly regulate the expression of target genes (Kim et al., 2006). However, our knowledge of the genes directly regulated by ANT/AILs remains limited. One known target gene of AtAIL2 is ACTIN DEPOLYMERIZING FACTOR9 (*ADF9*), which modulates somatic cell proliferation (Passarinho et al., 2008). The DAP-seq technique is a method used to identify transcription factor binding elements, initially validated by analyzing the binding targets of 529 transcription factors in *Arabidopsis thaliana* (O’Malley et al., 2016; Bartlett et al., 2017). Subsequently, DAP-seq has been applied in numerous species to identify the regulatory networks and target genes of transcription factors, including GmJAG1, DSM12444, ZMIBH1-1, MeGI, MwMYB-1, and PagLBD21 (Cao et al., 2020; Mai et al., 2023; Liu et al., 2024; Wang et al., 2024; Myers et al., 2025). In this study, we similarly employed DAP-seq to identify the target genes and regulatory network of GhAIL6. Results revealed GhAIL6 to also be involved in the MAPK signaling pathway. The MAPK cascade consists of three tiers of protein kinases: MAPK kinase kinase (MKKK), MAPK kinase (MKK), and MAPK (Xu and Zhang, 2015). *AtMKK4/5* acts as upstream of MAPK6 to regulate embryogenesis (Zhang et al., 2017). In rice, *OsMKKK10*, *OsMKK4*, and *OsMAPK6* function as a cascade to regulate seed size (Xu et al., 2018). We hypothesize that GhAIL6 utilizes the EAR motif to recruit GhTPL1, forming a transcriptional repressor complex that represses the expression of genes in the MAPK signaling pathway, thereby regulating seed index and lint percentage development.

Overall, using proteome data from 1,509 species, we clarified that the expansion of the ANT family occurred in the ancestor of angiosperms, with gene number tending to remain stable within the classes Monocots and Eudicots. However, ANT protein length has come to vary over the evolution of these two classes. This study offers novel insights into the evolutionary dynamics of ANT family members and length. Edited lines of GhAIL6 and GhTPL1, lint percentage was significantly higher than in the control group, and seed index significantly lower than in the control group. Finally, using the DAP-seq technique, we identified regulatory pathways and direct target genes downstream of GhAIL6. These target genes mediate the multiple functions of GhAIL6 in cotton development. Future studies remain necessary to further define the regulatory network of GhAIL6 and explore its roles in other biological processes.

## Data Availability

The datasets presented in this study can be found in the National Center for Biotechnology Information, accession number PRJNA1371402.
