# Supplementary material for: Evolution of ANT family and regulation of lint percentage by the GhAIL6-GhTPL1 module
Source: Front Plant Sci. 2025 Dec 5;16:1723288. doi: 10.3389/fpls.2025.1723288 (PMC12715526; doi:10.3389/fpls.2025.1723288)
Supplement: Supplementary file 1 [file Supplementaryfile1.docx]

Supplementary material

SUPPLEMENTARY FIGURE 1

The structure of GhAIL6^LLB^ and GhAIL6^HLB^. **(A)** Three-dimensional structure of GhAIL6LLB protein and the local spatial structure of the 175th amino acid. **(B)** Three-dimensional structure of GhAIL6LLB protein and the local spatial structure of the 185th amino acid. **(C)** Three-dimensional structure of GhAIL6HLB protein and the local spatial structure of the 175th amino acid. **(D)** Three-dimensional structure of GhAIL6HLB protein and the local spatial structure of the 185th amino acid. Red dots and circles represent the positions of amino acids in the spatial structure.

SUPPLEMENTARY FIGURE 2

Agarose gel electrophoresis detection of cotton genomic DNA. **(A)** Agarose gel electrophoresis detection of upland cotton genomic DNA. Lane M：DNA Marker 1kb； Lane 1: Upland cotton genomic DNA. **(B)** Fragmentation electrophoresis detection of genomic DNA from upland cotton. Lane M：DNA Marker 2000，GoldBand DL600 DNA Marker； Lane 1-5: Fragmentation of genomic DNA from upland cotton. **(C)** Quality analysis of expressed proteins. Lane M：180 kDa Prestained Protein Marker； Lane 1: GhAIL6 protein purified sample; Lane 2：0.5 mg/mL BSA. **(D)** Western-blot detection of expressed proteins. Lane 1: GhAIL6 protein purified sample; Lane 2：Multitag Protein. **(E)** GhAIL6 protein binding assay. Lane M：Protein Marker； Lane 1: Experimental group 1; Lane 2: Experimental group 2; Lane 3: control group; Lane 4：BSA.

SUPPLEMENTARY FIGURE 3

Peak length distribution of experimental group 1 (SYZ1) and experimental group 2 (SYZ2).


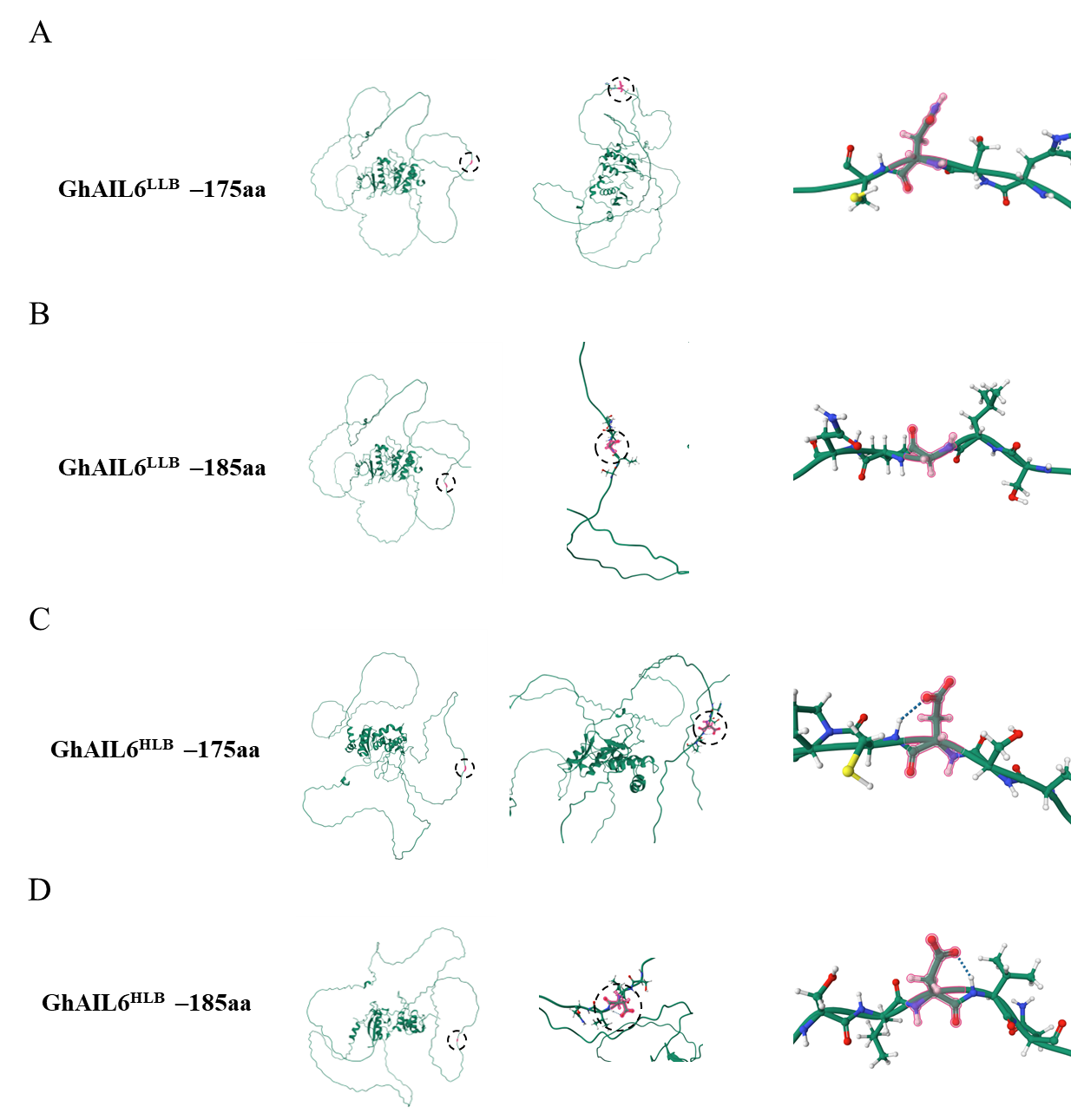


Supplementary Figure 1 The structure of GhAIL6^LLB^ and GhAIL6^HLB^. **(A)** Three-dimensional structure of GhAIL6^LLB^ protein and the local spatial structure of the 175th amino acid. **(B)** Three-dimensional structure of GhAIL6^LLB^ protein and the local spatial structure of the 185th amino acid. **(C)** Three-dimensional structure of GhAIL6^HLB^ protein and the local spatial structure of the 175th amino acid. **(D)** Three-dimensional structure of GhAIL6^HLB^ protein and the local spatial structure of the 185th amino acid. Red dots and circles represent the positions of amino acids in the spatial structure.


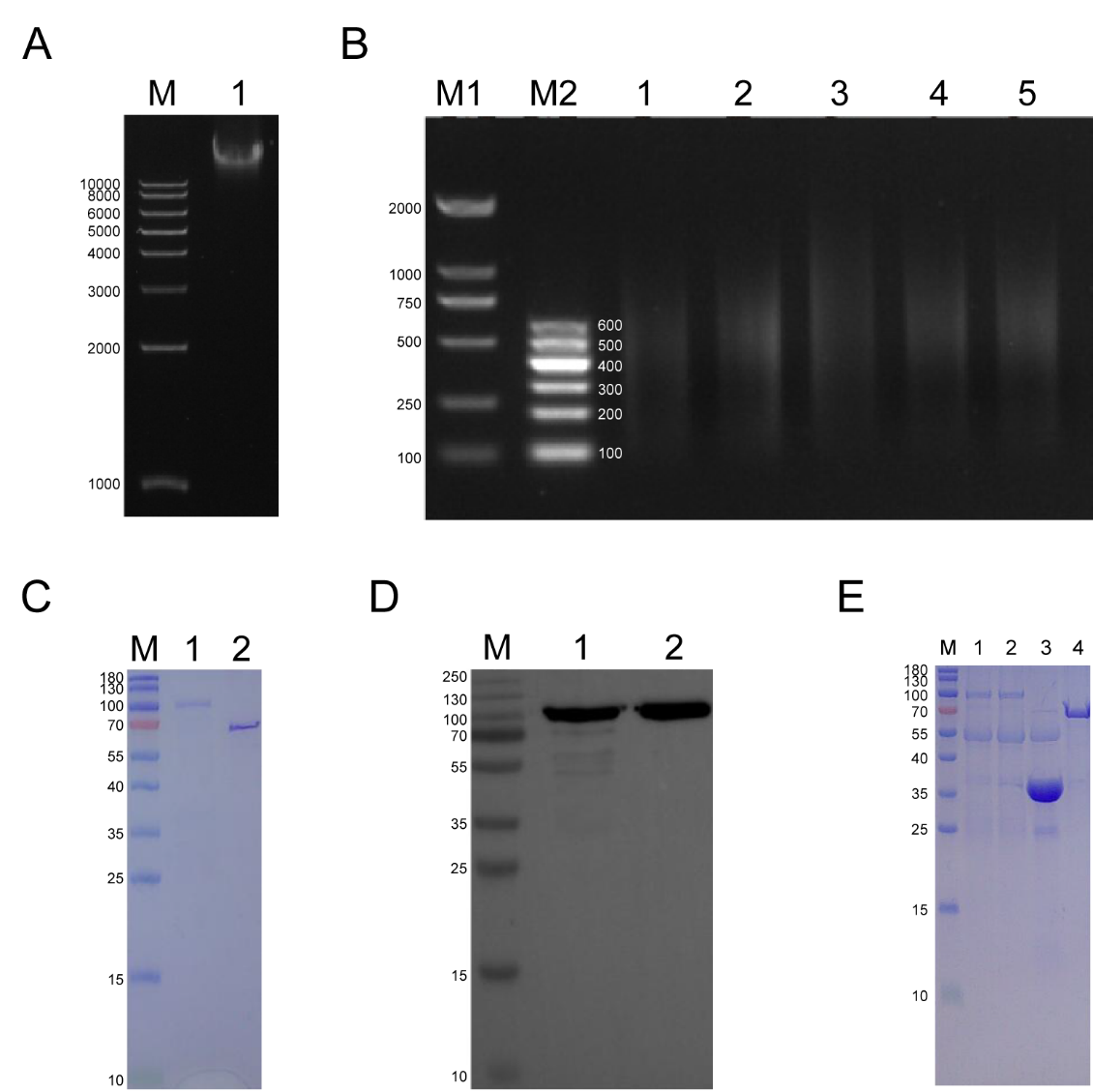


Supplementary Figure 2 Agarose gel electrophoresis detection of cotton genomic DNA. **(A)** Agarose gel electrophoresis detection of upland cotton genomic DNA. Lane M：DNA Marker 1kb； Lane 1: Upland cotton genomic DNA. **(B)** Fragmentation electrophoresis detection of genomic DNA from upland cotton. Lane M：DNA Marker 2000，GoldBand DL600 DNA Marker； Lane 1-5: Fragmentation of genomic DNA from upland cotton. **(C**) Quality analysis of expressed proteins. Lane M：180 kDa Prestained Protein Marker； Lane 1: GhAIL6 protein purified sample; Lane 2：0.5 mg/mL BSA。 **(D)** Western-blot detection of expressed proteins. Lane 1: GhAIL6 protein purified sample; Lane 2：Multitag Protein. **(E)** GhAIL6 protein binding assay. Lane M：Protein Marker； Lane 1: Experimental group 1; Lane 2: Experimental group 2; Lane 3: control group; Lane 4：BSA.


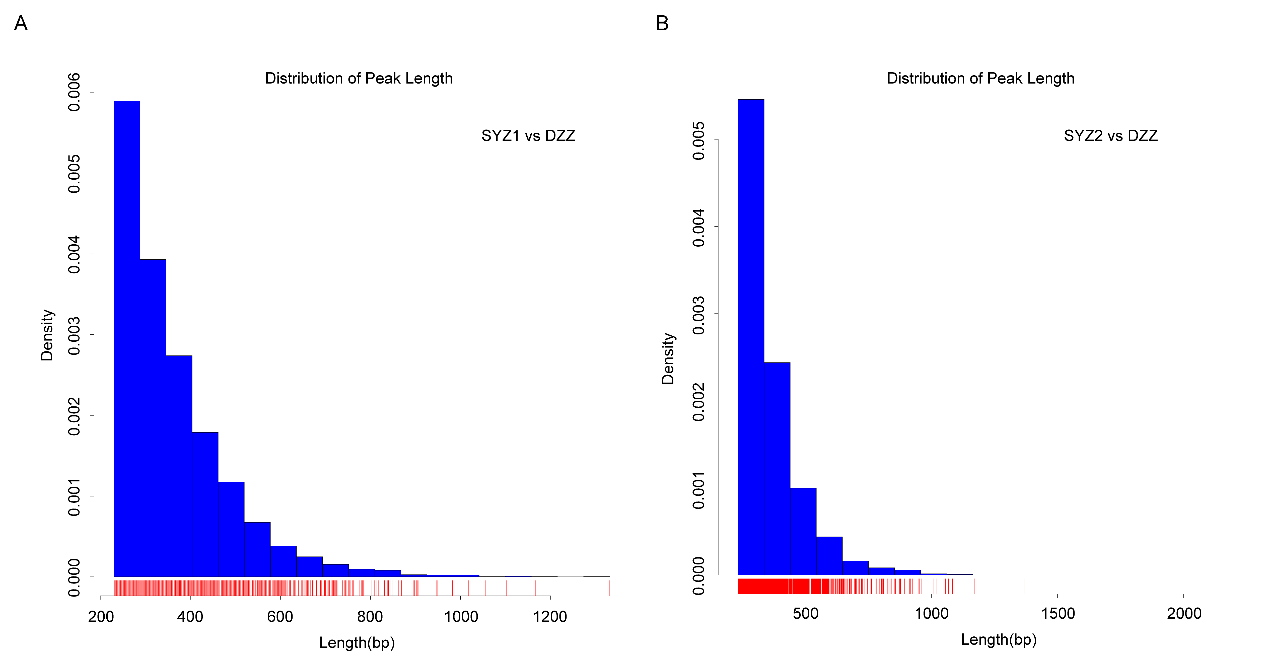


Supplementary Figure 3 Peak length distribution of experimental group 1 (SYZ1) and experimental group 2 (SYZ2).
